# Monocrotaline-induced liver toxicity in rat predicted by a combined in vitro physiologically based kinetic modeling approach

**DOI:** 10.1007/s00204-020-02798-z

**Published:** 2020-06-09

**Authors:** Suparmi Suparmi, Sebastiaan Wesseling, Ivonne M. C. M. Rietjens

**Affiliations:** 1 Division of Toxicology, Wageningen University and Research, Stippeneng 4, 6708 WE Wageningen, The Netherlands; 2 Department of Biology, Faculty of Medicine, Universitas Islam Sultan Agung, Jl. Raya Kaligawe KM 4, Semarang, 50112 Indonesia

**Keywords:** Monocrotaline, Acute toxicity, Liver, PBK modeling, Reverse dosimetry

## Abstract

The aim of the present study was to use an in vitro–in silico approach to predict the in vivo acute liver toxicity of monocrotaline and to characterize the influence of its metabolism on its relative toxic potency compared to lasiocarpine and riddelliine. In the absence of data on acute liver toxicity of monocrotaline upon oral exposure, the predicted dose–response curve for acute liver toxicity in rats and the resulting benchmark dose lower and upper confidence limits for 10% effect (BMDL_10_ and BMDU_10_) were compared to data obtained in studies with intraperitoneal or subcutaneous dosing regimens. This indicated the predicted BMDL_10_ value to be in line with the no-observed-adverse-effect levels (NOAELs) derived from availabe in vivo studies. The predicted BMDL_10_–BMDU_10_ of 1.1–4.9 mg/kg bw/day also matched the oral dose range of 1–3 mg PA/kg bw/day at which adverse effects in human are reported. A comparison to the oral toxicity of the related pyrrolizidine alkaloids (PAs) lasiocarpine and riddelliine revealed that, although in the rat hepatocytes monocrotaline was less toxic than lasiocarpine and riddelliine, due to its relatively inefficient clearance, its in vivo acute liver toxicity was predicted to be comparable. It is concluded that the combined in vitro-PBK modeling approach can provide insight in monocrotaline-induced acute liver toxicity in rats, thereby filling existing gaps in the database on PA toxicity. Furthermore, the results reveal that the kinetic and metabolic properties of PAs can vary substantially and should be taken into account when considering differences in relative potency between different PAs.

**Electronic supplementary material:**

The online version of this article (10.1007/s00204-020-02798-z) contains supplementary material, which is available to authorized users.

## Introduction

**Fig. 1:**
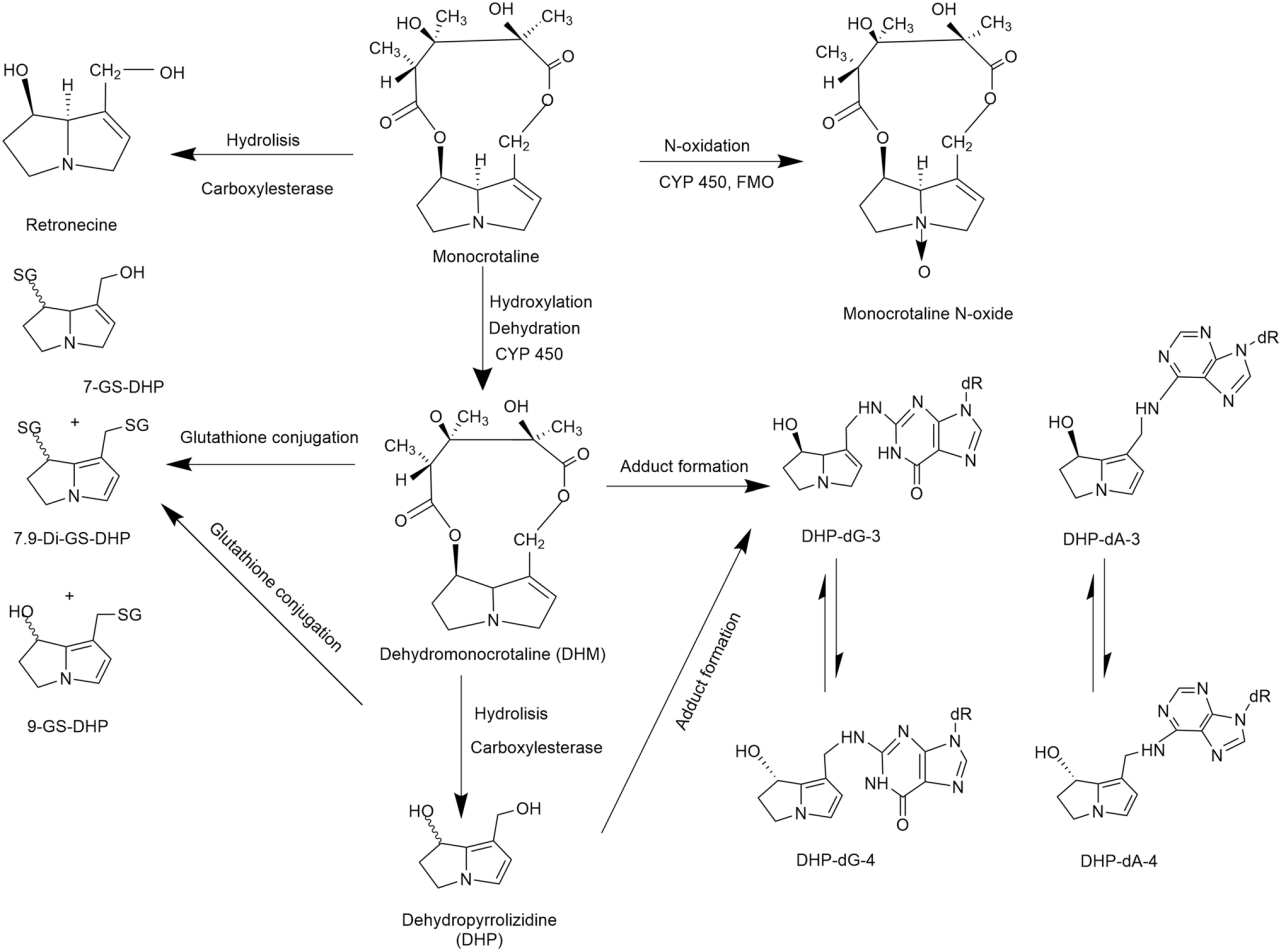
Schematic diagram of the metabolic pathways of monocrotaline and DNA adduct formation by monocrotaline metabolites relevant for rat and human (Wang et al. 2009; Xia et al. 2018; Yang et al. 2017; Yao et al. 2014). FMO = flavin-containing monooxygenase, CYP 450 = cytochromes P450

Monocrotaline (Fig. 1) is a secondary metabolite that belongs to a group of cyclic di-ester 1,2-unsaturated pyrrolizidine alkaloids (PAs). It is naturally present in Crotalaria species including *Crotalaria spectabilis*, *C. sagittalis* L., *C. retusa* L., and *C. aegyptiaca* Beth (Adams and Rogers 1939; EFSA 2011)*.* High acute toxicity of monocrotaline towards animals and humans has been reported (Copple et al. 2002, 2004; Lachant et al. 2018; Yan and Huxtable 1995). Recently EFSA (2017) listed monocrotaline as one of the 17 PAs to be monitored for their presence in food and feed because of possible concern for human health related to exposure to these PAs via food including consumption of tea and herbal infusions. PAs including monocrotaline are of concern because of their hepatotoxicity and the fact that they are genotoxic carcinogens (EFSA 2017). Monocrotaline is categorized as being possibly carcinogenic in humans (category 2B) (IARC 2019).

Like all 1,2-unsaturated PAs monocrotaline is a pro-toxin (unreactive compound) requiring hepatic metabolic activation by cytochromes P450 to exert hepatic toxicity and carcinogenicity (Mattocks 1968; Shumaker et al. 1976). CYP2A6 and CYP2E1 were found to be the major P450s active in metabolic activation of monocrotaline in rat and human liver (Ruan et al. 2014; Yao et al. 2014). The expression of CYP2A6 in human liver accounts for ∼4% of total hepatic CYPs, whereas in rats, the CYP2A family including CYP2A6 accounts for 2% (Martignoni et al. 2006). Also, the species differences in CYP2A6 activity between rats and human liver microsomes in inducing liver toxicity have been reported to be negligible (Pearce et al. 1992). Furthermore, the expression and activity of CYP2E1 in human have been reported to be 80% identical to that in rat (Martignoni et al. 2006), and, therefore, rats may be an appropriate model to study CYP2E1-dependent metabolism in human (Zuber et al. 2002). This is further supported by the fact that rats and human liver microsomes displayed qualitatively similar activation and detoxification activities in incubations with monocrotaline (Couet et al. 1996; Geburek et al. 2019). Metabolism of PAs is generally occurring via three pathways, namely hydrolysis, N-oxidation, and hydroxylation followed by dehydrogenation (Fig. 1) (Fu et al. 2004). Upon this dehydrogenation, an unstable and highly reactive intermediate, named dehydromonocrotaline is formed. Dehydromonocrotaline can react with cellular macromolecules including proteins and DNA to form protein and DNA adducts, which are considered to be responsible for the toxicity including the genotoxicity of monocrotaline (Butler et al. 1970; Lafranconi and Huxtable 1984; Reid et al. 1998). Alternatively, dehydromonocrotaline can be detoxified through hydrolysis resulting in 6,7-dihydro-7-hydroxy-1-hydroxymethyl-5*H*-pyrrolizidine (DHP) and via glutathione (GSH) conjugation resulting in formation of GSH-DHP and di-GSH-DHP (Fig. 1). These molecules are considered less toxic and more stable (Fu et al. 2004; Li et al. 2016), although they may still also react with proteins and DNA to form the same DNA adducts formed by dehydromonocrotaline and DHP (Xia et al. 2018).

Upon bioactivation, monocrotaline causes a variety of toxic insults including pulmonary endothelial apoptosis, acute lung injury, pulmonary fibrosis, necrotizing pulmonary arteritis, myocarditis, hepatic veno-occlusive disease (HVOD), pulmonary hypertension, and right ventricular hypertrophy (Fu 2017; Gomez-Arroyo et al. 2012; Li et al. 2011; Lu et al. 2018; Schultze and Roth 1998; Shumaker et al. 1976), in addition to an increased risk of developing liver carcinomas (Newberne and Rogers 1973). In human, acute exposure to PAs can cause HVOD with severe liver damage with in some cases fatal outcomes (Mohabbat et al. 1976; Tandon et al. 1976), whereas chronic exposure is considered to increase the risk of developing cancer (EFSA 2017).

However, only for a limited number of 1,2-unsaturated PAs in vivo toxicity data are available, and this implies that alternative testing strategies including read across and in vitro to in vivo extrapolation (IVIVE) become important. In previous studies, we reported the development and evaluation of physiologically based kinetic (PBK) models for the PAs lasiocarpine and riddelliine for rat and human, and their use for conversion of in vitro data for toxicity in primary hepatocytes to quantitatively predict in vivo acute liver toxicity for both rat and human (Chen et al. 2018; Ning et al. 2019). Marked differences in toxicokinetics were observed between these two PAs influencing the predicted in vivo toxicity. This importance of toxicokinetics in the relative differences in toxic potency between different PAs was also noted in a recent study that characterized the intrinsic relative potency of a series of PAs showing a role for the rate and extent of their metabolism (Lester et al. 2019). The aim of the present study was to use the in vitro-PBK model-facilitated reverse dosimetry approach to predict the in vivo acute liver toxicity of monocrotaline and to characterize the influence of its metabolism on its relative toxic potency compared to lasiocarpine and riddelliine. Monocrotaline was selected as the model compound because this is one of the few PAs in addition to lasiocarpine and riddelliine for which in vivo data on kinetics and liver toxicity are available, thus enabling evaluations of the PBK model and predictions made.

## Materials and methods

### Chemicals and biological materials

Monocrotaline (> 98%) was purchased from MedChemExpress (Huissen, The Netherlands). The plateable cryopreserved male rat (Sprague–Dawley) hepatocytes (RTCP10™), the thawing and plating supplement (serum-containing, CM 3000) pack, the cell maintenance supplement pack (serum free, CM4000), and Williams E Medium without phenol red (WEM, A1217601) were purchased from ThermoFisher (Naarden, The Netherlands). Pooled liver and intestinal microsomes from male Sprague–Dawley rats were purchased from Xenotech (Lenexa, USA). Dimethyl sulfoxide (DMSO) was obtained from Acros Organics (Geel, Belgium). Acetonitrile (UPLC/MS grade) was obtained from Biosolve (Valkenswaard, The Netherlands). Potassium hydrogen phosphate (K_2_HPO_4_) and trifluoroacetic acid (TFA) were purchased from Merck (Darmstadt, Germany). Fetal calf serum (FCS) and the reduced form of β-nicotinamide adenine dinucleotidephosphate sodium salt hydrate (NADPH) were obtained from Sigma-Aldrich (Zwijndrecht, The Netherlands). WST-1 (4-[3-(4-iodophenyl)-2-(4-nitrophenyl)-2H-5-tetrazolio]-1,3-benzene disulfonate) solution was purchased from Roche (Woerden, The Netherlands). Rapid equilibrium dialysis (RED) devices were purchased from Thermo Fisher Scientific (Bleiswijk, The Netherlands). Phosphate-buffered saline (PBS) was obtained from Invitrogen (Breda, The Netherlands).

### Outline of the PBK modeling-facilitated reverse dosimetry approach

The prediction of in vivo monocrotaline-induced liver toxicity in rat using a combined in vitro-PBK modeling approach consisted of the following steps: (1) establishment of an in vitro concentration–response curve for the toxicity of monocrotaline in primary rat hepatocytes, (2) development of a PBK model describing in vivo kinetics of monocrotaline, using kinetic parameters defined based on in vitro assays using rat liver and intestinal samples, (3) evaluation of the PBK model predictions against available literature data on dose-dependent blood levels of monocrotaline, (4) translation of the in vitro concentration–response curve for acute liver toxicity into an in vivo dose–response curve for acute liver toxicity in rat using the PBK model, taking into acount differences in protein binding of monocrotaline in the in vitro and in vivo situation, (5) benchmark dose (BMD) analysis on the predicted in vivo dose–response data to obtain a point of departure (POD), and (6) evaluation of the predicted POD for liver toxicity against available literature data.

### In vitro liver toxicity assay with primary rat hepatocytes

The monocrotaline-induced liver toxicity was tested in vitro using the WST-1 assay which measures the formazan formation by the metabolically active cells from WST-1. Pooled cryopreserved plateable male rat (Sprague–Dawley) hepatocytes (RTCP10™) were thawed and seeded in accordance with the manufacturer’s protocol. Briefly, cells were seeded in 96-well plates (Greiner bio-one, Alphen aan den Rijn, The Netherlands) at a concentration of 5 × 10^5^ cells/ml to give 1.25 × 10^4^ cells/well and incubated at 37 °C, 5% CO_2_ in a humidified atmosphere for 6 h to allow cell adherence. After incubation, medium was aspirated and then replaced by 100 µl/well of exposure medium (serum free) containing the required concentration of monocrotaline. The cells were incubated for 24 h at increasing concentrations (0–600 μM) of monocrotaline in exposure medium added from 200 times concentrated stock solutions in DMSO. The solvent DMSO (0.5% (v/v) final concentration in exposure medium) was used as a negative control and triton X (final concentration 1% (v/v) in exposure medium) served as a positive control in all cytotoxicity assays. After exposure for 24 h, 5 μl (1:20 dilution) WST-1 reagent was added to each well and plates were incubated for an additional 1 h. Then, the plate was shaken at 1000 rpm for 1 min, and absorbance was measured at 440 nm (background absorbance at 620 nm was subtracted) using a SpectraMax M2 (Molecular Devices, Sunnyvale, USA).

Data are presented as mean values ± SE from three independent experiments with three different batches of rat hepatocytes. The cell viability was expressed as percentage of the solvent control, with the solvent control set at 100%. The obtained concentration–response curves for hepatotoxicity were fitted with a symmetrical sigmoidal model (Hill slope) which was further used to derive IC_50_ values using log [inhibitor] vs. normalized response using GraphPad Prism software (version 5.00 for Windows, GraphPad software, San Diego, USA).

### In vitro incubations of monocrotaline with rat liver and intestinal microsomes to derive the kinetic parameters for the PBK model

The kinetic parameters for the PBK model of monocrotaline in rats were estimated by a substrate depletion approach using the protocol for microsomal incubations reported by Wang et al. (2009) with little modifications. The liver microsomal incubations were carried out in a total volume of 100 μl containing 0.1 M K_2_HPO_4_ (pH 7.4), 0.5 mg protein/ml of pooled rat liver/ intestinal microsomes, and monocrotaline at final concentrations ranging from 0 to 500 µM (added from 100 times concentrated stock solutions in 0.1 M HCl, the latter in line with the protocol of Wang et al. (2009), and shown to have no effect on the incubation pH). After 5 min of pre-incubation in a shaking water bath at 37 °C, the reactions were started by the addition of 1 mM NADPH. The reactions were carried out for 1 h and 2 h for liver and intestinal microsomes, respectively. For each incubation, a corresponding control incubated in the absence of NADPH was included by adding buffer instead of NADPH. To stop the metabolic conversion, 100 µl of ice-cold methanol was added and the sample was put on ice, then centrifuged at 5000 × g for 20 min at 4 °C using a microcentrifuge (CT15RE, VWR, Leuven, Belgium). Supernatants were diluted 200 times in 90% (v/v) acetonitrile and transferred to LC–MS vials. LC–MS analysis was performed using a Shimadzu Nexera XR LC-20AD SR UPLC system in tandem with a Shimadzu LCMS-8040 mass spectrometer (Shimadzu, Kyoto, Japan). From each incubation, 1 µl of supernatant was loaded onto a Luna Omega polar C18 100A LC column (1.6 µm 100 × 2.1 mm, Phenomenex) fitted with a FP polar precolumn (Phenomenex), using a flow rate of 0.3 ml/min. The temperature was set at 40 °C and 5 °C for column and sample, respectively. The mobile phase consisted of ultrapure water (solvent A) and acetonitrile (solvent B) both containing 0.1% (v/v) formic acid. The gradient began with 100% solvent A (0% B) for 1 min to wash away unwanted salts, followed by a linear gradient from 0 to 5% B till 8 min and a further increase to 100% B in 2 min, keeping the elution at 100% B for 0.5 min, then the column was set back to the starting conditions and equlibrated for 3.4 min before the next injection. The concentration of monocrotaline in the samples was quantified using a calibration curve prepared using a commercially available standard. For all incubations, three independent replicates were performed.

The time-dependent decrease in the concentration of monocrotaline detected in NADPH-containing reaction mixtures corrected for the time-dependent decrease in the concentration of monocrotaline in the corresponding controls without the cofactor NADPH was used to determine the rate of monocrotaline depletion. The data for the monocrotaline concentration-dependent rate of monocrotaline depletion thus obtained were fitted to the standard Michaelis–Menten equation (Eq. 1) using GraphPad Prism, 5.0 software (San Diego, CA, USA).

1 $$V = \frac{{V_{{\max}} \times \left[ S \right]}}{{\left( {K_{{\text{m}}} + \left[ S \right]} \right)}},$$

with [*S*] representing the monocrotaline concentration, *V*_max_ being the apparent maximum velocity (nmol/min/mg microsomal protein), and *K*_m_ being the apparent Michaelis–Menten constant (μM). The ratio of *V*_max_ and *K*_m_ was calculated as the in vitro catalytic efficiency (*k*_cat_) expressed in nmol/min/g tissue. The rat microsomal protein yield of 35 mg microsomal protein/g tissue and 20.6 mg microsomal protein/g tissue for liver and small intestine, respectively (Cubitt et al. 2009; Medinsky et al. 1994) were used to scale *V*_max_ and *k*_cat_ values obtained from the in vitro microsomal incubations to in vivo *V*_max_ and *k*_cat_ values expressed in nmol/min/g tissue and ml/min/g tissue, respectively. The rat liver weight of 8.5 g and small intestine weight of 3.5 g (see Table 1) (Brown et al. 1997) were used to scaled the in vivo *k*_cat_ values to values expressed in ml/min/tissue.

**Table 1:** Physiological and physicochemical parameters for rats applied in the PBK model for monocrotaline, lasiocarpine and riddelliine

Parameters	Symbol	Value
**Physiological parameters (Brown et al.**^1997^**)**		
Body weight (kg)	BW	0.25
Tissue volume (fraction of body weight)		
Fat	VFc	0.07
Liver	VLc	0.034
Small intestine	VSic	0.014
Blood	VBc	0.074
Richly perfused tissue	VRc	0.042
Slowly perfused tissue	VSc	0.75
Cardiac output (L/h/kg^0.74^)	QC	15
Blood flow to tissue (fraction of cardiac output)		
Fat	QFc	0.07
Liver	QLc	0.132
Small intestine	QSic	0.118
Richly perfused tissue	QRc	0.51
Slowly perfused tissue	QSc	0.17
**Physicochemical parameters (DeJongh et al. **1997**)**		
Tissue/blood partition coefficients		
Monocrotaline		
Fat	PF	0.46
Liver	PL	0.77
Small intestine	PI	0.77
Richly perfused tissue	PR	0.77
Slowly perfused tissue	PS	0.42
Lasiocarpine (Chen et al. 2018)		
Fat	PF	2.44
Liver	PL	0.88
Small intestine	PI	0.88
Richly perfused tissue	PR	0.88
Slowly perfused tissue	PS	0.48
Riddelliine (Chen et al. 2018)		
Fat	PF	0.44
Liver	PL	0.77
Small intestine	PI	0.77
Richly perfused tissue	PR	0.77
Slowly perfused tissue	PS	0.43

### Determination of fraction unbound (*f*_ub_) of monocrotaline in rat serum and correction for protein binding

The monocrotaline-induced liver toxicity is assumed to be dependent on the concentration of unbound monocrotaline available for bioactivation. To correct for the difference in protein binding in the in vitro incubations and the in vivo situation, the fraction unbound (*f*_ub_) of monocrotaline in the in vitro and in vivo situations was determined. Since the in vitro toxicity was determined in serum-free assay medium, the concentrations of monocrotaline tested were considered to be equal to the unbound concentration in the assay (*f*_ub,in vitro_ = 1.0). The f_ub,in vivo_ was determined by rapid equilibrium dialysis (RED) (Waters et al. 2008). Briefly, 200 µl of spiked rat serum containing 150 µM monocrotaline (final concentration, 0.5% v/v DMSO) was added to the serum chambers of the RED device insert, while 350 µl dialysis buffer (PBS) was added to the buffer chamber. The device was sealed with tape and incubated at 37 °C on a shaker at 250 rpm. After incubation for 5 h when the system reached equilibrium (van Liempd et al. 2011), 50 µl of post-dialysis samples was collected from the serum and buffer chambers into separate eppendorf tubes. Subsequently, 50 µl of rat serum was added to the buffer samples and 50 µl of PBS was added to the serum samples. To precipitate the protein, 300 µl of ice-cold acetonitrile (90% v/v) was added to both tubes. After putting the mixtures on ice for 30 min, the mixtures were centrifuged at 15,000 g for 30 min at 4 °C, and the supernatants were diluted five times in 90% (v/v) acetonitrile and analyzed by LC–MS as described above. The measurements were performed in triplicate.

The concentration of monocrotaline detected in each chamber was used to calculate *f*_ub,in vivo_ using Eq. 2 (van Liempd et al. 2011; Waters et al. 2008). The value of *f*_ub, in vivo_ was used in the PBK modelling-based reverse dosimetry to calculate the total concentration of monocrotaline in rat liver blood according to the Eq. 3.

2 $$f_{{{\text{ub, in vivo}} }} = \frac{{C_{{\text{b}}} }}{{C_{{\text{s}}} }},$$

3 $$C_{{{\text{monocrotaline, rat blood}} }} = \frac{{C_{{\text{ub, in vitro }}} }}{{f_{{\text{ub, in vivo}}} }},$$

where *f*_ub,in vivo_ represents the fraction unbound of monocrotaline in rat serum, *C*_b_ is the concentration of monocrotaline in the buffer chamber (μM), *C*_s_ is the concentration of monocrotaline detected in the serum chamber (μM), *C*_monocrotaline, rat blood_ is the total concentration of monocrotaline in rat blood (µM), *C*_ub,in vitro_ is the unbound concentration of monocrotaline in the in vitro culture medium which in the present study equals the concentration tested because *f*_ub,in vitro_ equals 1.0.

### Development and evaluation of a PBK model for monocrotaline in rat

**Fig. 2:**
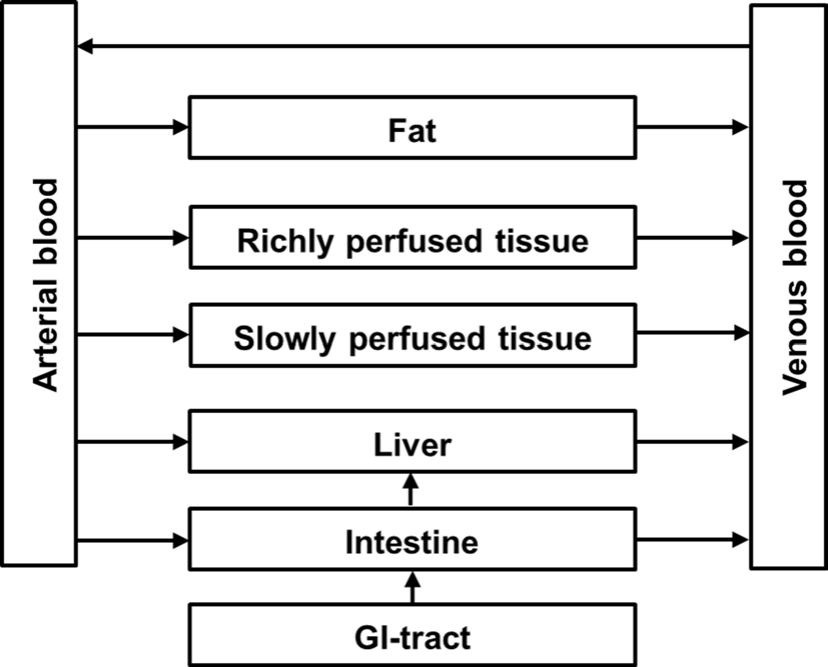
Schematic diagram of the PBK model for monocrotaline in rat, based on the model previously developed for lasiocarpine and riddelliine (Chen et al. 2018)

A PBK model for monocrotaline in rat was developed based on the models for lasiocarpine and riddelliine in rats (Chen et al. 2018). Figure 2 depicts the conceptual PBK model, which consists of seven separate compartments connected via the blood circulation. The physiological and anatomical parameters for rats were obtained from literature (Brown et al. 1997), while the blood/tissue partition coefficients for monocrotaline were estimated using the formula reported by (DeJongh et al. 1997) based on the water/octanol partition coefficient (log Kow) of monocrotaline of −0.65 predicted by ChemDraw 18.1 (Perkin-Elmer, USA) as presented in Table 1.

The absorption rate for uptake from the GI tract compartment into the liver (Ka) of monocrotaline was estimated based on the reported Ka for adonifoline (Wang et al. 2011), using the correlation of Caco-2 permeation and molecular properties described in literature (Hou et al. 2004), as follows:

4 $$\log P_{{{\text{app}}}} = - 5.469 + 0.236\log P,$$

5 $${\text{Ka}}_{{{\text{monocrotaline}}}} = { }\frac{{\log P_{{\text{app monocrotaline}}} { } \times {\text{ Ka}}_{{\text{ adonifoline }}} { }}}{{\log P_{{\text{app adonifoline }}} }},$$

where log *P*_app_ is the log value of the permeability coefficient (*P*_app_), log*P* is the water/octanol partition coefficient predicted by ChemDraw 18.1 (Perkin-Elmer, USA) being −0.65 and −1.49 for monocrotaline and adonifoline, respectively. The log *P*_app monocrotaline_ and log*P*_app adonifoline_ calculated by Eq. 4 are −5.62 for monocrotaline and −5.82 for adonifoline. Using the reported Ka for adonifoline of 0.6/h, the value of Ka for monocrotaline derived from Eq. 5 is 0.58/h. This Ka value was assumed to reflect efficient uptake of monocrotaline via passive diffusion. Tu et al. (2013) reported that the organic cation transporter 1 (OCT1) plays a role in active transport of monocrotaline into the liver. The substrates of OCT1 are known to be mainly organic cations, while some weak bases, non-charged compounds and anions are also transported (Koepsell and Endou 2004). Retronecine-type PAs including monocrotaline and retrorsine were also shown to be high affinity substrates of OCT1 (Tu et al. 2013,2014). At low pH, where monocrotaline is protonated to its corresponding cation transport by OCT1 is dominant and passive diffusion is almost abolished (Tu et al. 2013). At higher pH values in the intestinal compartment (Evans et al. 1988; McConnell et al. 2008), a substantial part of monocrotaline will be neutral and transported via passive diffusion. The data of Tu et al. (2013) indicate that in rat hepatocytes at pH 7.4, OCT1-mediated transport and passive diffusion may contribute equally. Thus, to take this OCT1-mediated transport into account, the Ka value as obtained from Eq. 5 for passive diffusion was multiplied by 2 to include the extra uptake via OCT1.

Furthermore, the data presented by Tu et al. (2013) provide an overall rate for uptake of monocrotaline into primary rat hepatocytes amounting to 14.5 pmol/mg protein/min at 2 µM monocrotaline and pH 7.4. Using scaling factors of 120 mg protein/g liver and 34 g liver/kg bw results in a rate for uptake of monocrotaline in the liver of rats of 3.55 µmol/h/kg bw. Using the Ka value of 1.16/h at a dose level of 1 mg/kg bw and the formula for uptake into the liver now used in the PBK model: ka (in /h) × AGI (in µmol/h/kg bw) results in a rate for uptake into the liver that equals 3.57 µmol/h/kg bw. This further supports that the use of the Ka of 1.16/h adequately models the overall uptake of monocrotaline in the liver.

The model code in Berkeley Madonna (version 9.1.14, UC Berkeley, CA, USA) using Rosenbrock’s algorithms for stiff systems for the developed PBK models of monocrotaline in rats is presented in supplementary materials 1. In the PBK model, the excretion of monocrotaline into urine was not included due to the fact that the excretion of monocrotaline as a parent compound in urine is negligible (Bull et al. 1968).

### Evaluation of the PBK model

To evaluate the PBK model performance, predicted monocrotaline concentrations in blood were compared to reported concentrations of monocrotaline equivalents in rat blood upon intravenous (iv) injection of 60 mg/kg bw (10 µCi/kg) of [^14^C] monocrotaline (Estep et al. 1991). To this end, the predicted time-dependent monocrotaline concentration in blood was compared to the time-dependent monocrotaline equivalent concentration curve reported by Estep et al. (1991) which was derived from the published curve of monocrotaline equivalents (in nmol/g) against time (in h) using webPlotDigitizer (https://automeris.io/WebPlotDigitizer/) under the assumption that the weight of blood plasma (g) is equal to the volume of blood (mL), because the blood density was assumed to be 1.0 g/ml. The final concentration of monocrotaline equivalents (µM) in whole blood was obtained by added up the concentration values in plasma and in red blood cells (supplementary materials 2) (Estep et al. 1991).

In addition, a sensitivity analysis was performed to identify the key parameters which contribute most to the predicted maximum concentrations in liver blood at an oral dose of 1 and 3 mg/kg bw which represent the lowest and highest dose in the range for the estimated daily human intake of PAs reported by EFSA (2017) that might result in adverse health effects if consumed for 4 days up to a 2-week periods.

The sensitivity analysis was performed as described previously (Evans and Andersen 2000) calculating normalized sensitivity coefficients (SCs):

6 $${\text{SC}} = \frac{{\left( {C^{\prime} - C} \right)}}{{P^{\prime} - P}} \times \left( \frac{P}{C} \right),$$

where *C* is the initial value of the model output, *C*′ is the modified value of the model output resulting from an increase in parameter value, *P* is the initial parameter value and *P*′ is the modified parameter value. Each parameter was analyzed individually by changing one parameter at a time (5% increase) and keeping the other parameters the same (Evans and Andersen 2000).

### Translation of in vitro liver toxicity to in vivo liver toxicity

The in vitro concentration–response curve for monocrotaline-induced cytotoxicity in primary rat hepatocytes was translated into a predicted in vivo dose–response curve for acute liver toxicity using PBK modeling-facilitated reverse dosimetry. Within this translation, a correction was made to take the difference in protein binding between the in vitro incubations (*f*_ub,in vitro_ = 1.00) and the in vivo situation (*f*_ub,in vivo_ determined as described above) into account. This was done because it was assumed that only the free fraction of monocrotaline will be available to be bioactivated and exert the effects. Each concentration tested in the cytotoxicity assay, corrected by Eq. 3 to calculate the corresponding total blood concentration, taking differences in in vitro and in vivo protein binding into acount, was set equal to the maximum concentration of monocrotaline in the liver blood and the developed PBK model was used to determine the coresponding oral dose. The dose–response curve for monocrotaline-induced liver toxicity resulting from this translation was compared to the previous predicted dose–response curves for lasiocarpine and riddelliine (Chen et al. 2018).

### BMD analysis of in vitro concentration–response data and of predicted in vivo dose–response data

To define the benchmark dose resulting in a 10% increase in liver toxicity over the background level (BMD_10_), the predicted in vivo dose–response data for monocrotaline-induced acute liver toxicity in rats were used for BMD modeling. To compare the toxic potency of monocrotaline with that of lasiocarpine and riddelliine, the predicted dose–response curves reported previously for these PAs (Chen et al. 2018) was also used for BMD modeling. Dose–response modeling and BMD analysis were performed using the EFSA BMD modeling webtool (PROAST version 66.38, https://shiny-efsa.openanalytics.eu/app/bmd) (EFSA-Scientific-Committee et al. 2017). The lowest Akaike Information Criterion (AIC) value among the available models was used to judge the the goodness of fit application of the models.

### Evaluation of the predicted POD for liver toxicity against available literature data

The predicted BMDL_10_–BMDU_10_ values of monocrotaline in this study were compared to the PODs derived from in vivo rat acute liver toxicity data on monocrotaline reported in the literature (Copple et al. 2002, 2004; Lachant et al. 2018; Yan and Huxtable 1996). When the data from these in vivo studies were not suitable for BMD analysis due to the limited number of data points and/or insufficient distribution of the data points over the dose–response curves, the no observed adverse effect level (NOAEL) was used for the comparison. When only a lowest observed adverse effect level (LOAEL) was available, the NOAEL was calculated using the LOAEL divided by a factor of 10 (Barnes et al. 1988).

## Results

### Monocrotaline-induced liver toxicity in vitro

**Fig. 3:**
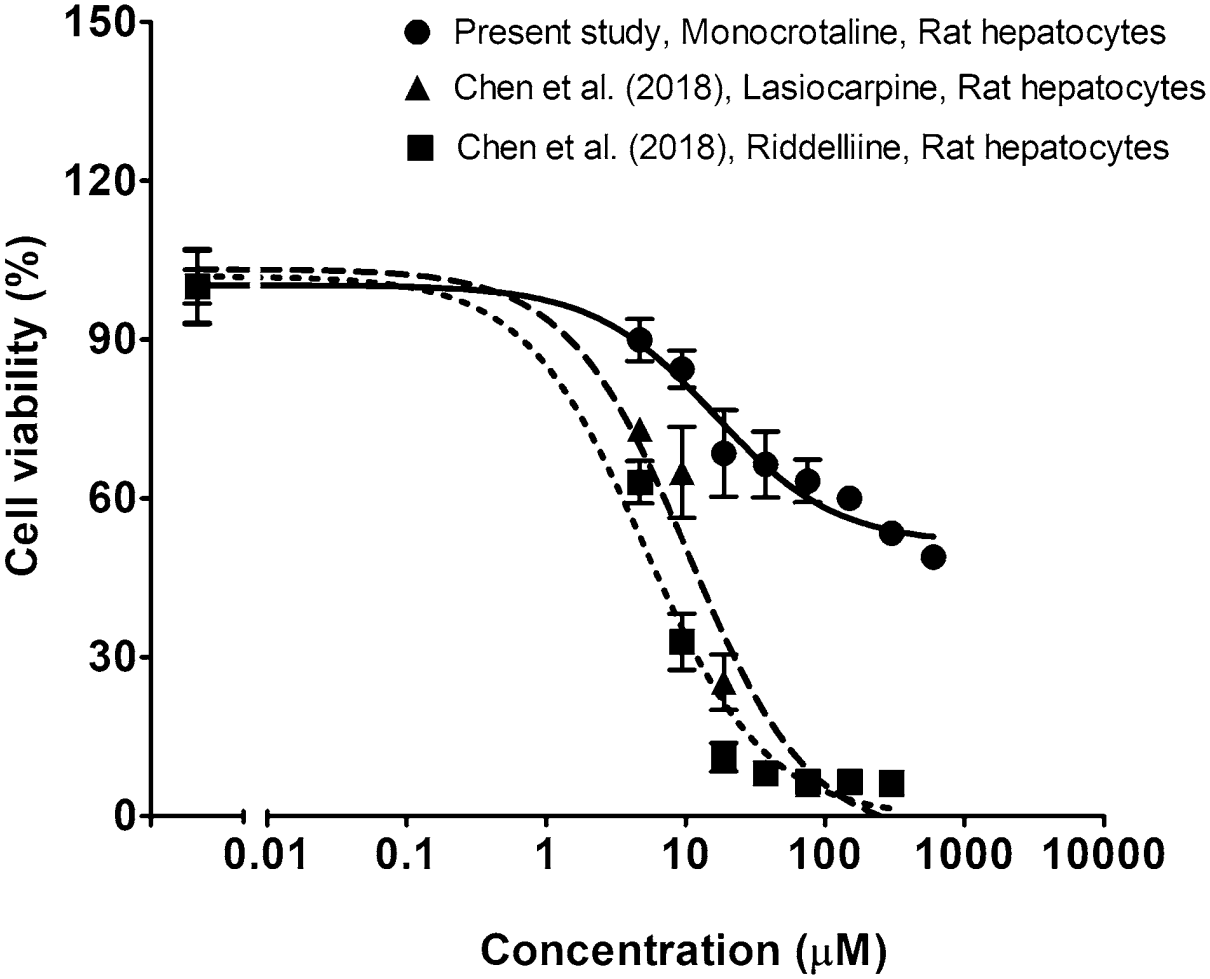
Concentration–response curves for effects of monocrotaline (circles with solid line) on cell viability of primary rat hepatocytes exposed for 24 h (means ± SE) and, for comparison, for effects of lasiocarpine (triangles with dashed line) and riddelliine (squares with dotted line) as reported by Chen et al. (2018)

Monocrotaline-induced liver toxicity in primary rat hepatocytes with an IC_50_ value of 225 µM as shown in Fig. 3. The highest concentration of 600 µM decreased cell viability by over 60% while limited solubility prevented testing of higher concentrations and reaching 100% cytotoxicity. The EC_50_ obtained for monocrotaline is 20.7- and 35.7-fold higher than the EC_50_ values previously obtained in the same model system for lasiocarpine (EC_50_ 10.9 μM) and riddelliine (EC_50_ 6.3 μM), respectively (Chen et al. 2018).

### Metabolic clearance of monocrotaline by rat liver and intestine microsomes

**Fig. 4:**
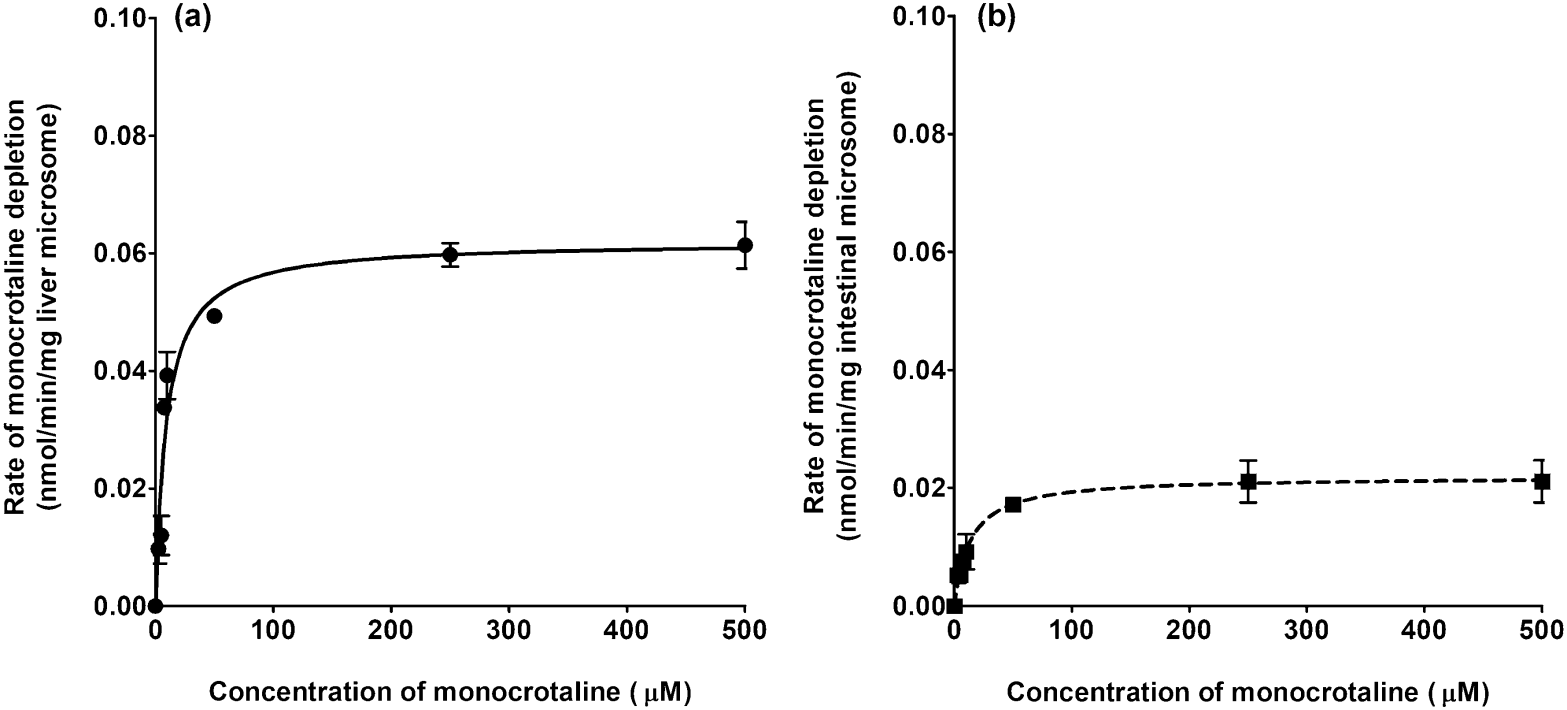
Concentration-dependent rate of monocrotaline depletion in incubations with: **a** rat liver microsomes and **b** intestinal microsomes. Values are presented as means ± SE derived from three independent experiments

Figure 4 shows the monocrotaline concentration-dependent rate of conversion of the compound in incubations with rat intestinal and liver microsomes. Table 2 presents the *V*_max_ and *K*_m_ values derived from these curves and also the catalytic effciency (*k*_cat_) for clearance of monocrotaline calculated as *V*_max_/*K*_m_. For comparison Table 2 also presents the kinetic parameters for depletion of lasiocarpine and riddelliine previously reported (Chen et al. 2018). It appears that monocrotaline is converted by the liver microsomes with an in vivo scaled *k*_cat_ (ml/min tissue) that is 18 times higher than the conversion rate by intestinal microsomes (Fig. 4 and Table 2). Lasiocarpine and riddelliine showed the same trend where the scaled catalytic efficiency for conversion expressed per intestinal tissue was 15.4 and 253 times, respectively, lower than that for the liver indicating the intestinal contribution to PA clearance to be minor (Table 2). The scaled *k*_cat_ for conversion of monocrotaline in the liver was 41.8 and 4.3 times lower compared to the scaled liver k_cat_ of lasiocarpine and riddelliine, respectively, indicating that the metabolism of monocrotaline was the lowest among the three PAs. The total scaled in vivo *k*_cat_ (sum of liver and intestine) for depletion of monocrotaline was 42.1- and 4.1-fold, respectively, lower than that for lasiocarpine and riddelliine.

**Table 2:** Kinetic parameters for metabolic conversion of monocrotaline (present study), lasiocarpine and riddelliine (Chen et al. 2018) in incubations with pooled rat liver and intestine microsomes

Compound Organ	*V*_max_ (nmol/min/mg microsomal protein)	*K*m (μM)	*k*_cat_ (ml/min/mg microsomal protein)	Scaled *V*_max_ (nmol/min/g tissue)^a^	Scaled *k*_cat_ (ml/min/g tissue)^a^	Scaled *k*_cat_ (ml/min/ tissue)^b^
Monocrotaline (present study)						
Liver	0.06	9.2	0.01	2.1	0.2	1.9
Intestine	0.02	13.4	0.001	0.4	0.03	0.1
Lasiocarpine (Chen et al. 2018)						
Liver	5.3	19.5	0.27	186	9.5	80.9
Intestine	1.7	23.4	0.07	35.0	1.50	5.2
Riddelliine (Chen et al. 2018)						
Liver	2.1	75.7	0.03	73.5	0.97	8.2
Intestine	0.1	221	0.0005	2.06	0.009	0.03

^a^Scaled *V*_max_ and *k*_cat_ calculated from the in vitro *V*_max_ and *k*_cat_ based on a microsome protein yield of 35 mg microsomal protein/(g liver) or 20.6 mg microsomal protein/(g small intestine) (Cubitt et al. 2009; Medinsky et al. 1994)

^b^Scaled in vivo *k*_cat_ (ml/min/ tissue) derived from the in vivo *k*_cat_ (ml/min/g tissue) based on the liver weight of 8.5 g or small intestine weight of 3.5 g (Brown et al. 1997)

### PBK model predictions and evaluation

Due to unavailability of in vivo kinetic data for monocrotaline upon oral administration in rat, the blood concentration–time curves of monocrotaline as predicted by the developed PBK model upon iv administration were evaluated against the available concentration of monocrotaline equivalents in rat blood upon the iv administration of 60 mg/ kg of [^14^C] monocrotaline (Estep et al. 1991). The predicted blood concentrations were on average 1.6- to 3.4-fold higher than the blood concentrations observed in vivo (see Table S1 supplementary materials 2). Given this limited deviation, it was concluded that the PBK model could be used for the in vitro to in vivo extrapolations.

### Sensitivity analysis

**Fig. 5:**
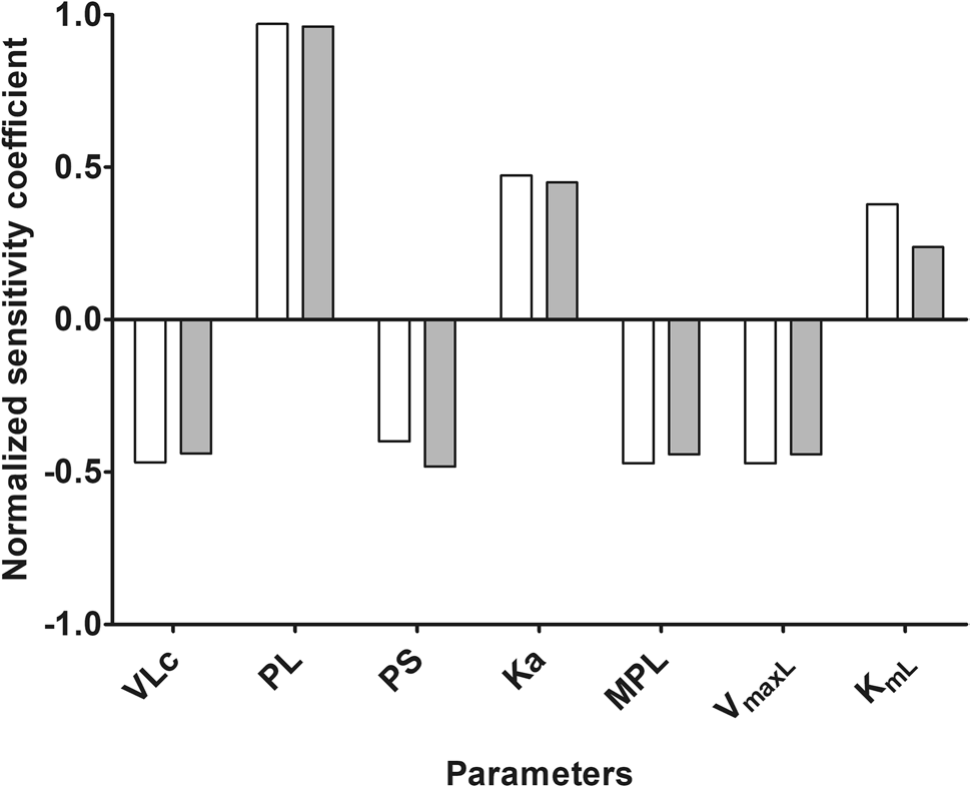
Normalized sensitivity coefficients for the parameters of the rat PBK model for monocrotaline of influence on the predicted maximum concentration in liver blood at a single oral dose of monocrotaline of 1 mg/kg bw (white bars), or 3 mg/kg bw/day (grey bars) PAs. VLc = fraction of liver volume, PL = liver/blood partition coefficient, PS = slowly perfused tissue/blood partition coefficient, Ka = absorption rate for uptake from the GI tract compartment into the liver, MPL = liver microsomal protein yield, *V*_maxL_ and *K*_mL_ = the maximum rate of depletion and the Michaelis–Menten constant for depletion of monocrotaline in liver

The performance of the developed PBK model was further evaluated by a sensitivity analysis to determine the parameters which affect the prediction of the maximum concentration of monocrotaline in liver blood. The parameters that result in a normalized sensitivity coefficient higher than an absolute value of 0.1 are shown in Fig. 5. At an oral dose level of of 1 and 3 mg/kg bw, representing the lowest and highest dose in the range for the estimated daily human intake of PAs that might result in adverse health effects if consumed for 4 days up to a 2 weeks periods (EFSA 2017), the predicted maximum concentration of monocrotaline in liver blood was affected by the fraction of liver volume (VLc), the partition coefficient of monocrotaline into liver tissue (PL), the partition coefficient into slowly perfused tissue (PS), the absorption rate from the GI tract compartment into the liver (Ka), the liver microsomal protein yield (MPL), and the kinetic parameters (*V*_maxL_ and *K*_mL_) for monocrotaline depletion in the liver. The predicted monocrotaline concentration in liver blood was not sensitive to the kinetic parameters for monocrotaline depletion in the small intestine-related parameters in line with the earlier observation that monocrotaline metabolism is this organ is substantially less efficient (Fig. 4).

### Predicted hepatotoxicity of monocrotaline in rats and application of PROAST modeling on predicted dose–response data to derive PODs

The RED assay resulted in an *f*_ub, in vivo_ of monocrotaline in rat serum of 0.53 ± 0.12, a value used to correct for the differences in protein binding between the in vivo and in vitro situation. With this *f*_ub,in vivo_, the concentrations tested in the cytotoxicity assay were converted to in vivo total blood concentrations by Eq. 3 and then converted to the corresponding dose levels using the PBK model. The dose levels thus obtained were used to create the corresponding dose–response curve for acute liver toxicity.

**Fig. 6:**
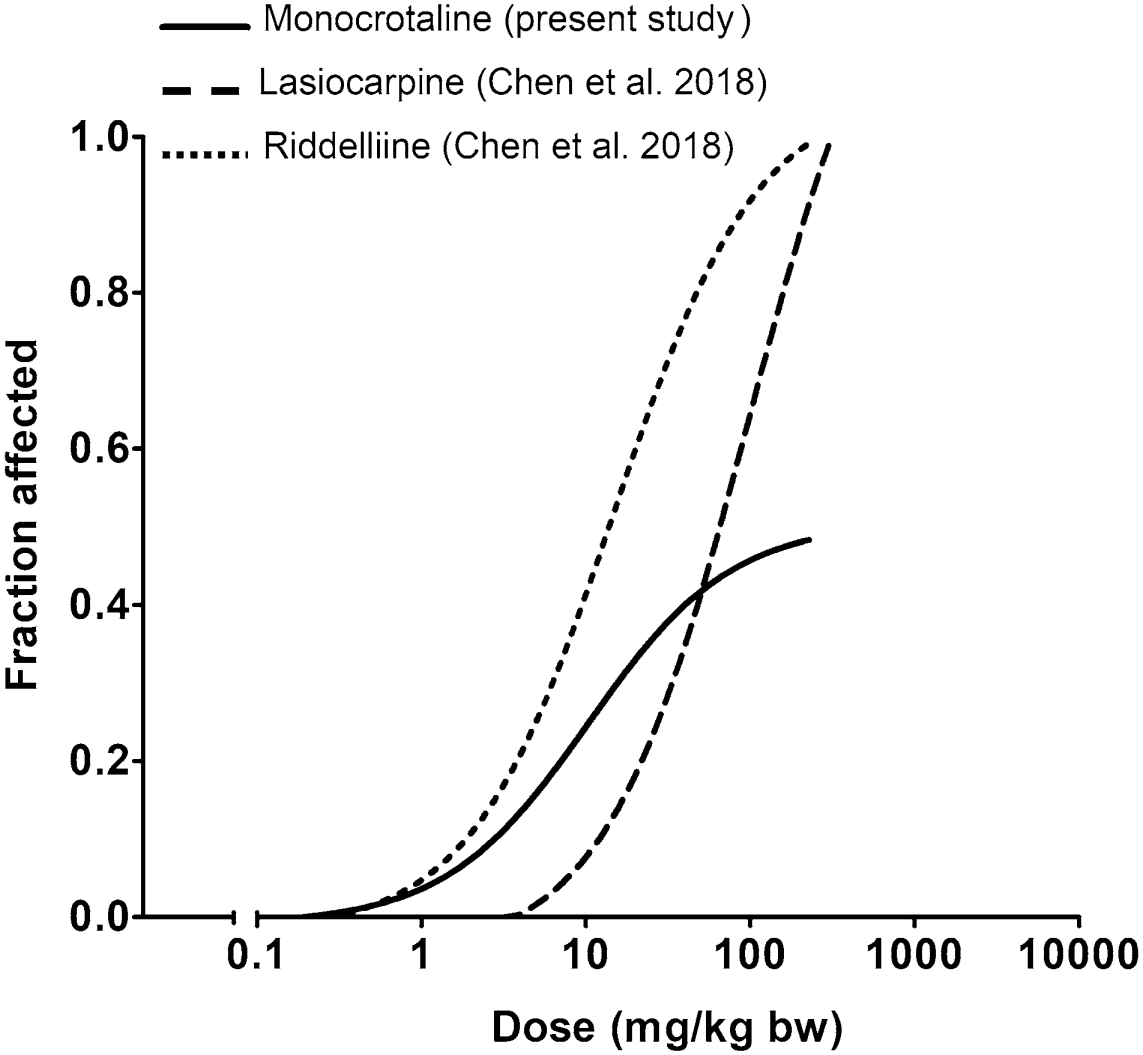
Predicted in vivo dose–response curves for acute liver toxicity in rats obtained by combining in vitro data in primary rat hepatocytes and PBK modeling-based reverse dosimetry for monocrotaline (black line). For comparison, the predicted dose–response curves for liver toxicity of lasiocarpine (red line) and riddelliine (blue line) reported by Chen et al. (2018) are also presented

The predicted in vivo dose–response curve thus obtained is shown in Fig. 6. For comparison, also the dose–response curves previously predicted for lasiocarpine and riddelliine by the same approach (Chen et al. 2018) are included in the figure. From the results obtained, it can be concluded that monocrotaline is predicted to be somewhat less toxic than riddelliine and somewhat more toxic than lasiocarpine. A BMD analysis was performed on the predicted dose–response data resulting in a predicted BMD_10_ and range of BMDL_10_–BMDU_10_ values for monocrotaline, lasiocarpine, and riddelliine as presented in Table 3. The predicted BMD_10_ for monocrotaline appeared to be 1.1-fold higher than that obtained from the predicted dose–response curve for riddelliine (Chen et al. 2018), while the value was 11.6 fold lower than that predicted for lasiocarpine.

**Table 3:** Predicted BMDL_10_–BMDU_10_ values derived from the dose–response curves presented in Fig. 6 predicted by PBK modeling-facilitated reverse dosimetry

Compound	Predicted BMDL_10_–BMDU_10_ (mg/kg bw/ day)	Predicted BMD_10_ (mg/kg bw/ day)	Source of the predicted dose–response curve
Monocrotaline	1.1–4.9	2.8	Present study
Riddelliine	1.3–3.7	2.6	(Chen et al. 2018)
Lasiocarpine	17.6–55.8	32.5	(Chen et al. 2018)

Comparison of these predicted differences in in vivo toxicity to the relative differences observed in vitro (Fig. 3) shows that the differences in in vivo toxicity between monocrotaline, lasiocarpine and riddelliine were subtantially different from the differences observed in vitro where lasiocarpine and riddelliine were 35.7 and 20.7, respectively, more toxic than monocrotaline. This shift towards relatively higher toxicity for monocrotaline in the in vivo situation is due to the differences in kinetics where monocrotaline appeared to be metabolised with a catalytic efficiency that was 42.1- and 4.1-fold lower than that for lasiocarpine and riddelliine, respectively. This implies that at similar dose levels, the accompanying blood concentrations and thus toxicity will be relatively higher for monocrotaline. This result corroborates that differences in kinetics substantialy influence the relative in vivo potencies of PAs, and should not be ignored when defining relative potency factors.

## Comparison of the predicted PODs to PODs derived from the reported data for liver toxicity in rats

To further evaluate the in vitro-PBK modeling-facilitated reverse dosimetry approach for prediction of monocrotaline-induced acute liver toxicity, the predicted BMDL_10_ for monoctotaline-induced liver toxicity was compared to the corresponding PODs (NOAEL values) derived from available in vivo studies for liver toxicity of monocrotaline in rats. Table 4 provides the overview of reported data on monocrotaline-induced acute liver toxicity in rats based on the endpoints of increased level of bound pyrrolic metabolites, increased alanine aminotransferase (ALT) activity, apoptosis of hepatic parencymal cells (HPC) and hepatic congestion (Copple et al. 2002, 2004; Lachant et al. 2018; Yan and Huxtable 1996). Since results from oral toxicity studies were not avalable, studies included in this comparison were studies with ip or sc dosing regimens. Given that the data of none of these studies enabled BMD modeling, the PODs from the available studies were based on the NOAEL or, when a NOAEL was not available, derived from the LOAEL value by assuming the NOAEL would amount to the LOAEL divided by 10 (Barnes et al. 1988) (Table 4).

**Table 4:** Monocrotaline-induced liver toxicity data reported for in vivo studies in male Sprague–Dawley rats

BW (g)	Exposure route	Dose (mg/kg bw/day) at single exposure	Effect	Type of POD	POD values (mg/kg bw)	Study
200–250	IP	0; 65	Increased level of bound pyrrolic metabolites levels 24 h after dosing	NOAEL (= LOAEL/10)	6.5	(Yan and Huxtable 1996)
100–130	IP	0; 100; 200; 225; 300	Increased plasma ALT 12 h after dosing	NOAEL	100	(Copple et al. 2002)
90–150	IP	0; 300	Apoptosis of HPC 18 h after dosing	NOAEL (= LOAEL/10)	30	(Copple et al. 2004)
> 200	SC	0;60	Hepatic congestion 24 h after dosing	NOAEL (= LOAEL/10)	6	(Lachant et al. 2018)

**Fig. 7:**
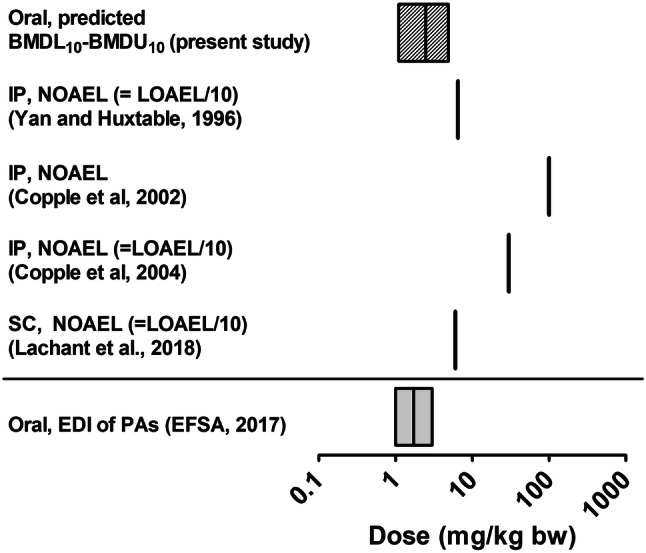
BMDL_10_–BMDU_10_ values for liver toxicity in rats predicted by the PBK modeling-facilitated reverse dosimetry approach using data for toxicity of monocrotaline in rat hepatocytes (patterned bar), compared to PODs derived from literature data on in vivo liver toxicity of monocrotaline in rats from studies with sc or ip dosing presented in Table 4 (vertical black bars). The grey bar below the line represents an oral dose range of 1–3 mg PA/kg bw/day at which acute/ short-term adverse effect in humans may occur (EFSA 2017)

Figure 7 presents a comparison of the predicted BMDL_10_–BMDU_10_ value of monocrotaline to the PODs data of Table 4. This comparison reveals that the reported toxicity data upon ip exposure vary substantially, and that the predicted BMDL_10_ value is in line with especially the NOAEL derived from the study with ip dosing reported by Yan and Huxtable (1996) and sc dosing reported by (Lachant et al. 2018). The BMDL_10_–BMDU_10_ of 1.1–4.9 mg/kg bw/day predicted by the in vitro–in silico approach of the present study is in line with the estimated toxic oral dose range of 1–3 mg PA/kg bw/day (EFSA 2017).

## Discussion

The aim of the present study was to use an in vitro–in silico approach to predict the in vivo acute liver toxicity of monocrotaline and to characterize the influence of its metabolism on its relative in vivo toxic potency compared to lasiocarpine and riddelliine. This in vitro–in silico approach was recently shown able to predict the acute liver toxicity of lasiocarpine and riddelliine (Chen et al. 2018). The results now obtained for monocrotaline further validate the approach as a possible method to fill existing gaps in the database on PAs relevant in food. Furthermore, comparison of the results to those previously obtained for lasiocarpine and riddelliine (Chen et al. 2018) corroborated the influence of metabolism on the relative toxic potency of these three PAs.

The in vitro concentration–response data for monocrotaline-induced toxicity were obtained using primary rat hepatocytes. Primary rat hepatocytes from pooled male Sprague–Dawley rats were used because male rats were previously reported to be more sensitive towards monocrotaline toxicity than female rats (Mattocks 1972) and also because most in vivo data available for the liver toxicity of monocrotaline were obtained in male rats (Table 4). Comparison of the in vitro toxicity data to the in vitro study of Louisse et al. (2019) which showed that monocrotaline did not exhibit cytotoxicity in HepaRG cells upon 24 h exposure, indicates that rat hepatocytes are more sensitive to the toxicity induced by monocrotaline. This result is in line with data from Ning et al. (2019) who reported that rat hepatocytes are more sensitive towards lasiocarpine and riddelliine induced liver toxicity than HepaRG cells. Primary rat hepatocytes likely contain higher levels of the cytochrome P450 enzymes required for metabolism including the bioactivation of parent PAs (Ruan et al. 2014; Yao et al. 2014). In the in vitro assay with rat primary hepatocytes, the IC_50_ value of monocroaline was 20.7- and 35.7-fold higher than the IC_50_ values previously reported in the same model system for lasiocarpine and riddelliine, respectively (Chen et al. 2018). The lower toxicity of monocrotaline in in vitro liver model systems is also in line with what has been observed in other studies using HepG2 or HepaRG cells (Kusuma et al. 2014; Louisse et al. 2019).

Since in the in vitro models used the liver toxicity of monocrotaline is quantified depending on the concentration of the parent compound, which is metabolised to its toxic metabolites within the cells of the in vitro model system, the PBK model developed in the present study describes the kinetics of monocrotaline and not of its metabolites and also the reverse dosimetry is based on concentrations of the parent compound. The substrate depletion analysis indicated that monocrotaline was slowly metabolized in the incubations with rat liver and intestinal microsomes. The kinetic efficiency for monocrotaline conversion appeared to be 42.1- and 4.1-fold lower compared to that previously obtained for lasiocarpine and riddelliine, respectively, using the same approach by Chen et al. (2018). This indicates that the metabolism of monocrotaline was the lowest among these three PAs. This result is line with the study performed by Lester et al. (2019) who reported that monocrotaline is metabolically stable in the rat sandwich culture hepatocyte cell system. Marked differences in metabolic degradation among PAs was also reported recently by Geburek et al. (2019) using in vitro incubations with rat liver microsomes indicating as well that conversion of monocrotaline was lower than that of riddelliine. In the present study, these kinetic differences were taken into account when translating the concentration–response curves for in vitro toxicity to the predicted dose–response curves for acute liver toxicity using PBK model-facilitated reverse dosimetry approach.

Evaluation of the developed PBK model for monocrotaline showed that the predicted concentrations of monocrotaline in blood were in line with the kinetic data available for monocrotaline in rats (Estep et al. 1991). The PBK model used was also similar to that previously developed and evaluated for the PAs lasiocarpine and riddelliine (Chen et al. 2018). Chen et al. (2018) demonstrated that the developed PBK model could adequately predict blood concentrations of riddelliine and also adequately translate the in vitro liver toxicity induced by lasiocarpine to a predicted in vivo dose–reponse curve for liver toxicity. The results of the present study reveal that the same approach can quantitatively predict the reported in vivo acute liver toxicity of monocrotaline. The predicted BMDL_10_ value appeared to be in line with the NOAELs derived from availabe in vivo studies, although the comparison also revealed that especially the NOAELs derived from the reported toxicity data upon ip exposure vary substantially, in part due to the fact that the NOAELs or LOAELs were the lowest dose levels tested, leaving room for the actual LOAEL and NOAEL being lower than what has now been derived from the data. The predicted BMDL_10_ was in line with the NOAEL derived from the study with sc dosing reported by Lachant et al. (2018). The differences observed may in part also be ascribed to the difference in dosing regimen with the predicted values refering to oral exposure, while the in vivo were from studies with ip or sc dosing. Due to the lack of data for monocrotaline-induced acute toxicity via oral intake in rats, the predicted BMDL_10_–BMDU_10_ value was also compared to the oral dose range of 1–3 mg PA/kg bw/day at which acute/ short-term adverse effects in human are reported to occur when consuming a combination of PAs via teas or herbal infusions (EFSA 2017). The BMDL_10_–BMDU_10_ of 1.1–4.9 mg/kg bw/day predicted by the in vitro–in silico approach of the present study is in line with this estimated toxic oral dose level, indicating that the toxicity of monocrotaline would match the overall toxicity estimated for PAs.

The result of the present study also indicated that taking the kinetics into account the predicted in vivo differences in toxicity between monocrotaline and lasiocarpine and riddelliine appeared to be smaller than what would be predicted based on the vitro data obtained in primary hepatocytes. Comparison of the in vitro and in vivo differences in toxicity between the three PAs reveals that differences in kinetics change the relative potencies in vivo compared to those detected in vitro. The sensitivity analysis of the PBK model elucidates which parameters and thus differences between the PAs contribute to this effect. In addition to differences in metabolic clearance (*V*_max_ and *K*_m_) also other sensitive parameters contribute. These include PL (liver/blood partition coefficient), PS (slowly perfused tissue/blood partition coefficient), and Ka (absorption rate for uptake from the GI tract compartment into the liver). All these parameters influence the maximum concentration (*C*_max_) of monocrotaline in liver blood (Fig. 5), thereby influencing the conversion of in vitro concentrations into in vivo dose levels and thus also the effect of taking kinetics into account when converting in vitro to in vivo relative potencies. The fact that a higher PL results in a higher *C*_max_ implies that for compounds with equal in vitro toxicity, a higher PL will result in a shift to relatively higher in vivo toxicity at similar dose level. Conversely, a higher PS results in relatively lower *C*_max_ values and relatively lower toxicity at a comparable dose level. In addition, a higher Ka value resulting in a higher absorption rate for uptake from the GI tract compartment into the liver, implies that a relatively lower dose level is required to reach a similar *C*_max_, so that toxicity will already be observed at lower dose levels.

Given that the sensitivity analysis ranked the partition coefficients as influential parameters on the model output, it is of interest to note that physicochemical properties, including lipophilicity, are reported to be important factors affecting the metabolic activation among PAs (Geburek et al. 2019; Mattocks 1981; Ruan et al. 2014). PAs with high lipophilicity (high log P values) resulted in relatively higher levels of reactive metabolites than observed for PAs with lower log P values. Using the ChemDraw 18.1 (Perkin-Elmer, USA), the log P values of lasiocarpine, riddelliine and monocrotaline amounted to 0.48, − 0.26 and − 0.65, respectively. The values of Ka derived from Eq. 5 for lasiocarpine, riddelliine and monocrotaline were 1.75/h, 1.17/h (Chen et al. 2018), and 0.58/h, respectively. The log Papp values and Ka values derived from these log *P* values, which were influential parameters in the PBK model, varied in line with the order of metabolic efficiency in incubations with rat liver and intestinal microsomes being lasiocarpine > riddelliine > monocrotaline.

The predicted BMDL_10_ value for acute liver toxicity of monocrotaline obtained in the present study supports the classification of monocrotaline as a toxic PA, with a potency for acute liver toxicity that seems comparable to that of lasiocarpine and riddelliine. To what extent this conclusions also holds for the carcinogenicity of these PAs remains to be established. The conclusion of similar potency is in line with the provisional relative potency factors (pRPF) derived by Merz and Schrenk (2016) indicating that monocrotaline, as well as riddelliine is categorized as one of the most potent congeners with a pRPF similar to that of lasiocarpine of 1.0. This result is in contrast to the ranking presented by Xia et al. (2013) based on the formation of DNA adducts, who ranked monocrotaline as group II with moderate tumour formation. Louisse et al. (2019) classified monocrotaline into group 3 with an pRPF of 0.06 based on its in vitro γH2AX induction potency in the human liver cell line HepaRG, while lasiocarpine and ridddelliine were categorized as group 1 with a pRPF of 1.08 and 1, respectively. However, these in vitro studies are based on different endpoint and also do not take potential differences in in vivo toxicokinetics into account, while the result of the present study clearly indicate that this will hamper the translation of in vitro RPFs to the in vivo situation. The lower metabolic clearance of monocrotaline than of lasiocarpine and riddelliine observed in the present study is in line with the results from Lester et al. (2019) and Geburek et al. (2019), and will result in higher relative in vivo concentrations and potential toxicity than predicted based on in vitro concentration–response curves.

In conclusion, the results of the present study illustrate that a combined in vitro–in silico approach can be used to obtain insights in monocrotaline-induced acute liver toxicity in rats. Furthermore, the comparison of its relative toxic potency to lasiocarpine and riddelliine indicates that the kinetic and metabolic properties of these PAs should be taken into account when defining relative differences in in vivo toxic potency. It is of relevance to note that the PBK model based reverse dosimetry was based on the parent compound. The predicted in vivo data obtained include both the relative differences in bioactivation (included in the in vitro toxicity data) and the relative differences in clearance of the parent compound (included via the PBK model). In the present study on monocrotaline, and our previous studies on lasiocarpine and riddelliine (Chen et al. 2018), the PBK model parameters for metabolic clearance were obtained using incubations with rat liver microsomes. Use of primary rat hepatocytes would have been an alternative and adequate approach but would less well match with our ultimate aim to contribute to the 3Rs (Replacement, Reduction and Refinement) for animal testing. This insight can be used to obtain a promising alternative testing strategy in risk and safety evaluation of PAs.

## Electronic supplementary material

Below is the link to the electronic supplementary material.

Supplementary file1 (DOCX 20 kb)

Supplementary file2 (DOCX 1241 kb)

## Abbreviations

AIC: Akaike information criterion
ALT: Alanine aminotransferase
BMD_10_: Benchmark dose resulting in a 10% effect above background level
BMDL_10_: Lower confidence limit of the BMD_10_
BMDU_10_: Upper confidence limit of the BMD_10_
EFSA: European Food Safety Authority
GI: Gastrointestinal
GSH: Glutathione
HPC: Hepatic parencymal cells
HVOD: Hepatic veno-occlusive disease
Ka: Absorption rate for uptake from the GI tract compartment into the liver
PAs: Pyrrolizidine alkaloids
PBK: Physiologically based kinetic
POD: Point of departure
NOAEL: No observed adverse effect level
LOAEL: Lowest observed adverse effect level
pRPF: Provisional relative potency factors
3Rs: Replacement, reduction and refinement
