# Supplementary material for: Monocrotaline-induced liver toxicity in rat predicted by a combined in vitro physiologically based kinetic modeling approach
Source: Arch Toxicol. 2020 Jun 9;94(9):3281–95. doi: 10.1007/s00204-020-02798-z (PMC7415757; doi:10.1007/s00204-020-02798-z)
Supplement: Supplementary file 1 — Supplementary file1 (DOCX 20 kb) [file 204_2020_2798_MOESM1_ESM.docx]

**Monocrotaline-induced liver toxicity in rat predicted by a combined in vitro-physiologically based kinetic modeling approach**

Suparmi Suparmi^1,2*^, Sebastiaan Wesseling^1^, Ivonne M.C.M. Rietjens^1^

^1^Division of Toxicology, Wageningen University and Research, Stippeneng 4, 6708 WE Wageningen, The Netherlands

^2^Department of Biology, Faculty of Medicine, Universitas Islam Sultan Agung, Jl. Raya Kaligawe KM 4, 50112 Semarang, Indonesia

***Corresponding author:**

Suparmi. Division of Toxicology, Wageningen University and Research

Stippeneng 4, 6708 WE Wageningen, The Netherlands

Tel: +31 317 84357

Fax: +31 317 484931

E-mail addresses: [s.suparmi@wur.nl](mailto:s.suparmi@wur.nl), suparmi@unissula.ac.id

Orcid ID: 0000-0001-7914-870X

**Supplementary materials 1. PBK model code for monocrotaline in rats**

; Purpose : Monocrotaline PBK model

; Species : Rats

;===============================================================================

;Physiological parameters

;===============================================================================

;Tissue volumes

BW = 0.250 {Kg} ; body weight rat (variable, dependent on study)

VFc = 0.07 ; fraction of fat tissue reference: (Brown et al., 1997)

VLc = 0.034 ; fraction of liver reference: (Brown et al., 1997)

VSic = 0.014 ; fraction of small intestine reference: (Brown et al., 1997)

VAc = 0.0185 ; fraction of arterial blood: 0.074*1/4 (calculated)

VVc = 0.0555 ; fraction of venous blood: 0.074*3/4 (calculated)

VBc = 0.074 ; fraction of blood reference: (Brown et al., 1997)

VRc = 0.09-VLc-VSic ; fraction of richly perfused tissue reference: (Brown et al., 1997)

VSc = 0.82-VFc ; fraction of blood flow to slowly perfused tissue (calculated)

VF = VFc*BW {L or kg} ; volume of fat tissue (calculated)

VL = VLc*BW {L or kg} ; volume of liver tissue (calculated)

VSi = VSic*BW {L or kg} ; volume of small intestine tissue (calculated)

VR = VRc*BW {L or kg} ; volume of richly perfused tissue (calculated)

VS = VSc*BW {L or kg} ; volume of slowly perfused tissue (calculated)

VA = VAc*BW {L or kg} ; volume of arterial blood (calculated)

VV = VVc*BW {L or kg} ; volume of venous blood (calculated)

VB = VBc*BW {L or kg} ; volume of blood (calculated

;--------------------------------------------------------------------------------------------------------------------------------------

;Blood flow rates

QC = 15*BW^0.74 {L/hr} ; cardiac output reference: (Brown et al., 1997)

QFc = 0.07 ; fraction of blood flow to fat reference: (Brown et al., 1997)

QLc = 0.25 -QSic ; fraction of blood flow to liver reference: (Brown et al., 1997)

QSic = 0.76*0.014/0.09 ; fraction of blood flow to small intestine reference: (Brown et al., 1997)

QRc = 0.76-QLc-QSic ; fraction of blood flow to richly perfused tissue reference: (Brown et al., 1997)

QSc = 0.24-QFc ; fraction of blood flow to slowly perfused tissue reference: (Brown et al., 1997)

QF = QFc*QC {L/hr} ; blood flow to fat tissue (calculated)

QL = QLc*QC {L/hr} ; blood flow to liver tissue (calculated)

QSi = QSic*QC {L/hr} ; blood flow to small intestine tissue (calculated)

QR = QRc*QC {L/hr} ; blood flow to richly perfused tissue (calculated)

QS = QSc*QC {L/hr} ; blood flow to slowly perfused tissue (calculated)

;===============================================================================

;Partition Coefficients

;===============================================================================

PL = 0.77 ; liver/blood partition coefficient calculated using QPPR of DeJongh et al. (1997)

PF = 0.46 ; fat/blood partition coefficient calculated using QPPR of DeJongh et al. (1997)

PR = 0.77 ; richly perfused tissues/blood partition coefficient calculated using QPPR of DeJongh et al. (1997)

PS = 0.42 ; slowly perfused tissues/blood partition coefficient calculated using QPPR of DeJongh et al.(1997)

PSi = 0.77 ; small intestine/blood partition coefficient calculated using QPPR of DeJongh et al. (1997)

;===============================================================================

;Biochemical parameters

;===============================================================================

;Linear uptake rate (hr-1)

Ka = 1.16 ; calculated from Papp derived from equation log Papp = -5.469 + 0.236 log P (Hou et al., 2004)

and multiplied by 2 to include the extra uptake via OCT1 (Tu et al. 2013)

;---------------------------------------------------------------------------------------------------------------------------------------

;Metabolism liver

;Scaling factors

MPL=35 {mg/g liver}; Liver microsomal protein yield reference: (Atio et al. 1976)

L=VLC*1000 {g/kg} ; Liver = 34

;metabolites of monocrotaline, unscaled maximum rate of metabolism (nmol/min-1/mg protein)

VmaxLM1c = 0.06 ;based on substrate depletion of monocrotaline by rat liver microsomes

;metabolites of monocrotaline, scaled maximum rate of metabolism (umol hr-1)

VMaxLM1 = VMaxLM1c/1000*60*MPL*L*BW

;metabolites of monocrotaline, affinity constants (umol/L)

KmLM1 = 9.22 ;based on substrate depletion of monocrotaline by rat liver microsomes

;---------------------------------------------------------------------------------------------------------------------------------------

;Metabolism small intestine

;Scaling factor

MPSi=20.6 {mg/g liver} ;Small intestine microsome fraction yield reference: (Cubitt et al. 2009)

Si =VSiC*1000

;metabolites of monocrotaline, unscaled maximum rate of metabolism (nmol/min-1/mg protein)

VmaxSiM2c = 0.02 ;based on the substrate depletion of monocrotaline by intestine microsomes

;metabolites of monocrotaline, scaled maximum rate of metabolism (umol hr-1)

VMaxSiM2 = VMaxSiM2c/1000*60*MPSi*Si*BW

;metabolites of monocrotaline, affinity constants (umol/L)

KmSiM2 = 13.39 ; based on substrate depletion of monocrotaline by intestine microsomes

;===============================================================================

;Run settings

;===============================================================================

;Molecular weight

MW =325.36; ;Molecular weight monocrotaline

;oral dose

ODOSEmg1 = 0 {mg/kg bw} ; ODOSEmg1 = given oral dose in mg/kg bw

ODOSEumol2 = ODOSEmg1*1E-3/MW*1E6 {umol/ kg bw} ;ODOSEumol2 = given oral dose recalculated to umol/kg bw

ODOSEumol = ODOSEumol2*BW; ; ODOSEumol = umol given oral

;--------------------------------------------------------------------------------------------------------------------------------------

;Intravenous (IV) dose

IVDOSEmg1 = 60 {mg/kg bw} ; IVDOSEmg1 = given IV dose in mg/kg bw

IVDOSEumol2 = IVDOSEmg1*1E-3/MW*1E6 {umol/ kg bw}

;IVDOSEumol2 = given oral dose recalculated to umol/kg bw

IVDOSEumol=IVDOSEumol2*BW ; IVDOSEumol = umol given IV

;time

Starttime = 0 ; in hr

Stoptime = 24 ; in hr

DTMIN = 1e-6 ; minimum integration time (DT)

DTMAX = 0.0015 ; maximum integration time (DT)

;===============================================================================

;Model calculations

;===============================================================================

;needle

;ANe = Amount monocrotaline in needle

ANe' = -kd*ANe

Init ANe = IVDOSEumol

kd=1000000 ;kd, the transport rate from needle to blood

;--------------------------------------------------------------------------------------------------------------------------------------

;===============================================================================

;Dynamics

;===============================================================================

;--------------------------------------------------------------------------------------------------------------------------------------

;uptake monocrotaline from GI tract

;AGI = Amount monocrotaline remaining in GI tract (umol)

AGI' =-ka*AGI

Init AGI = ODOSEumol

;-------------------------------------------------------------------------------------------------------------------------------------

;small intestine compartment

;ASi = Amount monocrotaline in small intestine tissue, umol

ASi' = QSi*(CB - CVSi) + ka*AGI - AMSiM2'

Init ASi = 0

CSi = ASi/VSi

CVSi = CSi/PSi

;AMSiM2=Amount monocrotaline metabolized in small intestine to metabolites, umol

AMSiM2'= VmaxSiM2*CVSi/(KmSiM2 + CVSi)

Init AMSiM2 = 0

;--------------------------------------------------------------------------------------------------------------------------------------

;liver compartment

;AL = Amount monocrotaline in liver tissue, umol

AL' = QL*CB +QSi*CVSi - (QL+QSi) *CVL - AMLM1'

Init AL = 0

CL = AL/VL

CVL = CL/PL

;AMLM1=Amount monocrotaline metabolized in liver to metabolites, umol

AMLM1' = VmaxLM1*CVL/(KmLM1 + CVL)

Init AMLM1 = 0

;--------------------------------------------------------------------------------------------------------------------------------------

;slowly perfused tissue compartment

;AS = Amount monocrotaline in slowly perfused tissue, umol

AS' = QS*(CB-CVS)

Init AS = 0

CS = AS/VS

CVS = CS/PS

;---------------------------------------------------------------------------------------------------------------------------------------

;richly perfused tissue compartment

;AR = Amount monocrotaline in richly perfused tissue, umol

AR' = QR*(CB-CVR)

Init AR = 0

CR = AR/VR

CVR = CR/PR

;---------------------------------------------------------------------------------------------------------------------------------------

;fat compartment

;AF = Amount monocrotaline in fat tissue, umol

AF' = QF*(CB-CVF)

Init AF = 0

CF = AF/VF

CVF = CF/PF

;---------------------------------------------------------------------------------------------------------------------------------------

; arterial blood compartment

;CA = Concentration monocrotaline in arterial blood

;CA = CV

;---------------------------------------------------------------------------------------------------------------------------------------

; venous blood compartment

;CB = Concentration monocrotaline in venous blood (umol/L)

AB' = (kd*ANe + QF*CVF + (QL+QSi)*CVL + QR*CVR + QS*CVS - QC*CB)

Init AB = 0

CB = AB/VB

AUC' = CB ;umol*min/L

init AUC = 0

;===============================================================================

;Mass balance calculations

{Mass Balance}

Total =ODOSEumol + IVDOSEumol

Calculated = AGI + ASi + AL + AS + AR + AF + AB + AMLM1 + AMSiM2 + ANe

ERROR=((Total-Calculated)/Total+1E-30)*100

MASSBBAL=Total-Calculated + 1

;===============================================================================

; blood concentration in ng/ml

CBngmL = CB*MW

;===============================================================================
