# Supplementary material for: Monocrotaline-induced liver toxicity in rat predicted by a combined in vitro physiologically based kinetic modeling approach
Source: Arch Toxicol. 2020 Jun 9;94(9):3281–95. doi: 10.1007/s00204-020-02798-z (PMC7415757; doi:10.1007/s00204-020-02798-z)
Supplement: Supplementary file 2 — Supplementary file2 (DOCX 1241 kb) [file 204_2020_2798_MOESM2_ESM.docx]

**Monocrotaline-induced liver toxicity in rat predicted by a combined in vitro-physiologically based kinetic modeling approach**

Suparmi Suparmi^1,2*^, Sebastiaan Wesseling^1^, Ivonne M.C.M. Rietjens^1^

^1^Division of Toxicology, Wageningen University and Research, Stippeneng 4, 6708 WE Wageningen, The Netherlands

^2^Department of Biology, Faculty of Medicine, Universitas Islam Sultan Agung, Jl. Raya Kaligawe KM 4, 50112 Semarang, Indonesia

***Corresponding author:**

Suparmi. Division of Toxicology, Wageningen University and Research

Stippeneng 4, 6708 WE Wageningen, The Netherlands

Tel: +31 317 84357

Fax: +31 317 484931

E-mail addresses: [s.suparmi@wur.nl](mailto:s.suparmi@wur.nl), suparmi@unissula.ac.id

Orcid ID: 0000-0001-7914-870X

**Supplementary materials 2. Data pharmacokinetic data of monocrotaline upon the IV administration of 60 mg/kg bw (10 µCi/kg) of [^14^C] monocrotaline (Estep et al. 1991)**


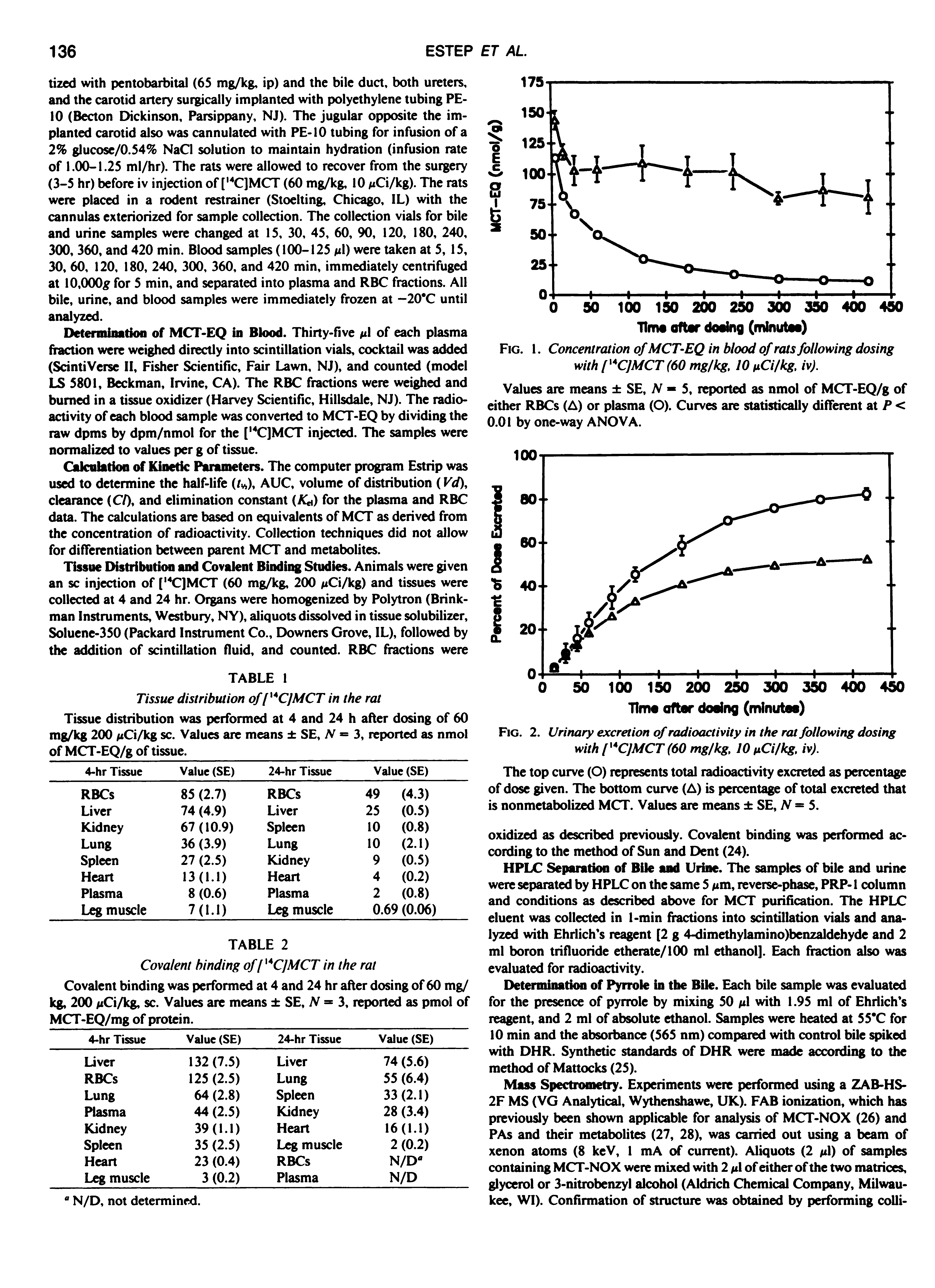


**Figure S1**. The screenshotted figure of monocrotaline equivalents (in nmol/g) against time (in h) reported by (Estep et al. 1991). The data symbolized as open circle (blood plasma) and triangle (red blood cells/RBCs) were added up to evaluate the concentration of monocrotaline in the whole blood for comparison to the PBK model predictions.

**Table S1. The results of re-extrapolated data from reported figure.**

In the PBK model, the volume of blood assumes to be equal to the weight of blood using this model

Monocrotaline equivalents (nmol/g) vs Time (min) data were created from the Figure S1 by WebPlotDigitizer Tutorial 3.6+ (https://automeris.io/WebPlotDigitizer/)

The reported concentration nmol/g is assumed to be equal to nmol/ml or µM. The reported blood concentration is calculated as the sum of the plasma and RBC concentration.

| Time for Blood sampling | | | Reported concentration of monocrotaline equivalents (µM) | | | | | | Predicted concentration of monocrotaline in blood (µM) | | Ratio of predicted/reported | |
| --- | --- | --- | --- | --- | --- | --- | --- | --- | --- | --- | --- | --- |
| min | h | | Plasma | | RBCs | | Blood | |  |  |  |  |
| 5 | 0.08 | 113.00 | | 144.00 | | 257.00 | | 416.50 | | 1.62 | |  |
| 15 | 0.25 | 81.88 | | 113.46 | | 195.34 | | 373.53 | | 1.91 | |  |
| 30 | 0.5 | 67.35 | | 100.06 | | 167.41 | | 370.88 | | 2.22 | |  |
| 60 | 1 | 49.84 | | 100.13 | | 149.97 | | 366.27 | | 2.44 | |  |
| 120 | 2 | 29.65 | | 104.89 | | 134.54 | | 357.08 | | 2.65 | |  |
| 180 | 3 | 21.82 | | 99.93 | | 121.75 | | 347.89 | | 2.86 | |  |
| 240 | 4 | 17.11 | | 99.60 | | 116.71 | | 338.70 | | 2.90 | |  |
| 300 | 5 | 13.07 | | 79.81 | | 92.88 | | 329.53 | | 3.55 | |  |
| 360 | 6 | 12.16 | | 85.50 | | 97.66 | | 320.36 | | 3.28 | |  |
| 420 | 7 | 11.00 | | 81.00 | | 92.00 | | 311.19 | | 3.38 | |  |

**Reference**

Estep JE, Lame MW, Morin D, Jones AD, Wilson DW, Segall HJ (1991) [^14^C]monocrotaline kinetics and metabolism in the rat. Drug Metab Dispos 19(1):135-9. <http://dmd.aspetjournals.org/content/19/1/135.long>
